# Respiratory Syncytial Virus Outbreak in Infants and Young Children during COVID-19 Pandemic in Taiwan

**DOI:** 10.3390/children10040629

**Published:** 2023-03-28

**Authors:** Hsin Chi, Ching-Hu Chung

**Affiliations:** 1 Department of Pediatrics, Mackay Memorial Hospital and Mackay Children’s Hospital, Taipei 104, Taiwan; 2 Department of Medicine, Mackay Medical College, New Taipei City 252, Taiwan

**Keywords:** respiratory syncytial virus (RSV), hospitalization, COVID-19

## Abstract

Respiratory syncytial virus (RSV) is a major burden of disease in babies and young children, including hospitalizations and deaths. RSV is a seasonal disease that peaks when temperatures decrease in temperate zones and humidity increases in tropical regions. Existing research reveals that RSV hospitalization activity is year-round in Taiwan, which is a subtropical region with small peaks in spring and fall. The monthly distribution and COVID-19 pandemic impact were unclear. The aim of this study was to investigate Taiwan’s RSV hospitalization seasonality and the COVID-19 pandemic effects. The National Health Insurance Database and Death Registration Files from the Center for Health and Welfare Data Science Center were connected to birth data for this study. RSV hospitalization (RSVH) in infants aged 0–1 years ranged from 0.9518% (2009) to 1.7113% (2020), substantially higher than in children aged 1–5. Most years had 2 or 3 RSV epidemic seasons in 0–5-year-olds over the 13-year follow-up. RSVH incidence was low until the autumn of 2020, when a major rise occurred after September and lasted until December 2020. We detected RSVH peaks in February–May and July–August. The 2020 RSV outbreak was found at the end of 2020.

## 1. Introduction

Respiratory syncytial virus (RSV) is linked with a high disease burden including hospitalizations and deaths of babies and young children [1]. Although RSV symptoms range from asymptomatic/mild to severe, young children are more likely to develop lower respiratory infections (LRTI) such as acute bronchiolitis and pneumonia [2,3]. The World Health Organization estimated that 63% of acute respiratory infections in children and more than 80% in infants younger than one year old are caused by RSV globally [4]. RSV also accounts for 50–80% of hospitalized acute bronchiolitis in North America, as well as almost 40% of severe pneumonia in developing countries [5,6]. In a single-center study in Taiwan in the 1990s, 32% of infants and young children admitted with LRTI were positive for RSV [7]. More recent results from 8 medical centers in Taiwan between November 2010 and September 2013, which was before the inclusion of pneumococcal vaccines in national immunization programs, showed that 4.9% of children and adolescents (aged 6 weeks to 18 years) hospitalized due to community-acquired pneumonia (CAP) were positive for RSV. RSV was the second leading virus causing CAP hospitalizations in young children under 2 years old, increasing to 17% [8].

Most of the children were reported to have been infected with RSV at the age of two [3]. For infants younger than one year old, RSV contributes a significant portion of mortality and morbidity: RSV bronchiolitis was a major cause of hospitalizations for babies under one year old, and RSV death was ten times higher than influenza [9,10]. Prematurity, congenital heart disease, and bronchopulmonary dysplasia (BPD) are risk factors for severe RSV diseases, but there is no reliable scoring method to identify which children will advance to catastrophic outcomes [5,11]. While the co-morbid groups are more prone to developing severe outcomes, more than two-thirds of the young children admitted due to RSV in the United States (US), and even up to 90% of young children hospitalized due to RSV in Taiwan, were born healthy and at term. Intensive care unit (ICU) admissions and mechanical ventilation usage also showed a similar trend, with healthy-term infants comprising around 60% of the RSV-related events in the US and 79.5% and 58.1%, respectively, in Taiwan [12,13,14,15,16].

RSV is a seasonal disease that peaks when temperate zone temperatures decrease and tropical humidity increases [17]. Previous studies in Taiwan, a subtropical country, demonstrated year-round RSV hospitalization activity with small peaks in spring and autumn [14,18,19]. The monthly distribution in central and southern Taiwan showed that only a fall peak existed [20,21]. The COVID-19 pandemic also influenced the worldwide RSV pattern, which has been demonstrated in the out-of-season outbreaks in 2020 and 2021 [20,22,23].

Few RSV studies have been published in Taiwan, and the most recent epidemiology study used data from before 2010 (with ICD-9 codes). To better look at the pattern of RSV and the COVID-19 effect, we utilized the National Health Insurance Research Database (NHIRD) with a retrospective analysis using recent data with ICD-9 and ICD-10 codes to evaluate the real-world burden of RSV in infants and young children from 2008 to 2020 in Taiwan.

## 2. Materials and Methods

### 2.1. Data Source

The Taiwan NHIRD included 23 million beneficiaries in Taiwan. By linking together several computerized claims datasets, we were able to use this database to make a long-term medical history for each beneficiary. The information on newborns was identified in the Maternal and Child Health Database and linked with the Birth Certificate Application Database through the identification number unique to each beneficiary. The study protocol was approved by the MacKay Memorial Hospital Institutional Review Board Taiwan, R.O.C. (Protocol Number: 17MMHIS114, approval date: 13 November 2017 until 12 November 2023).

### 2.2. Patient Selection

We examined the source population between 2008 and 2020. Patients were classified as having RSVH if they had a hospital admission record with one of the following diagnoses at discharge: Years 2008–2015, using ICD-9-CM codes 079.6 (RSV infection), 480.1 (RSV pneumonia), and 466.11 (RSV bronchiolitis). Years 2016–2020, using ICD-10-CM codes B97.4 (RSV as the cause of diseases classified elsewhere), J20.5 (acute bronchitis due to RSV), J21.0 (acute bronchiolitis due to RSV), and J12.1 (RSV pneumonia).

### 2.3. Data Analyses

RSVHs by month from 2008 to 2020 and their proportion among index years were recorded and estimated to compare seasonality. For monthly RSVH, the annual average percentage (AAP) was calculated to measure the RSV virus activity each year. The formula is AAPi = (number of RSVHs in index month/total number of RSVHs in index year) × 100%. The minimum number of months to accumulate for total AAP to reach 75% was estimated as the RSV epidemic season. This method was modified by You Li et al. [24]. SAS 9.4 (SAS Institute Inc., Cary, NC, USA) was used to analyze the data. Variable measures were identified using the above criteria, and categorical variables were described using frequencies or percentages.

## 3. Results

### 3.1. Annual Incidence of RSVH

**Figure 1:**
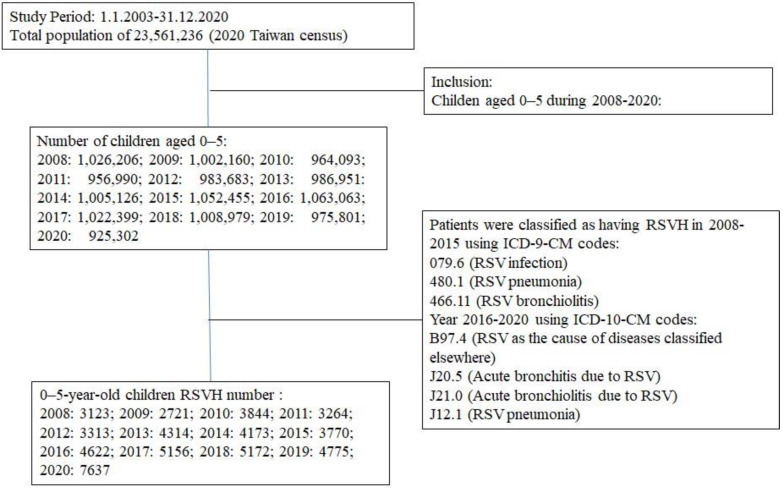
Enrolment flow chart.

**Figure 2:**
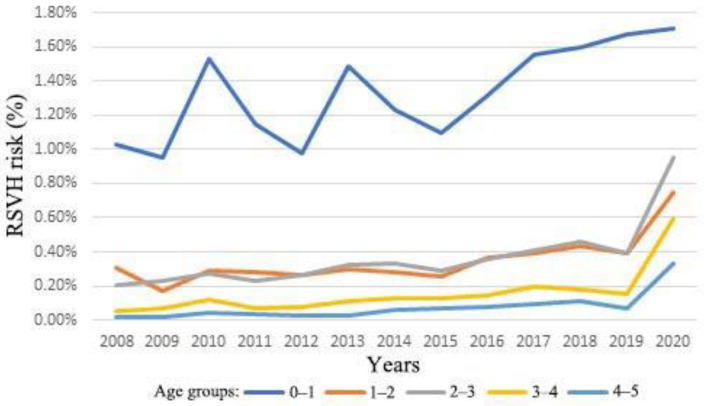
Annual incidence of RSVH among 0–5-year-old children. Children aged 0–5 between 2008 and 2020 were selected and classified as having RSVH if they had a hospital admission record with one of the following diagnoses at discharge: ICD-9-CM codes (years 2008–2015) 079.6 (RSV infection), 480.1 (RSV pneumonia), and 466.11 (RSV bronchiolitis); ICD-10-CM codes (years 2016–2020) B97.4 (RSV as the cause of diseases classified elsewhere), J20.5 (acute bronchitis due to RSV), J21.0 (acute bronchiolitis due to RSV), and J12.1 (RSV pneumonia). The RSVH risk was calculated by total number of RSVHs in index year divided by total number of children of index year.

Figure 1 displays the study inclusion and counts for cohort assignment. From 2008 to 2020, there were 925,302 to 1,063,063 0–5-year-old children included in this study, with 2721 to 7637 RSV hospitalizations (RSVHs) among these 0–5-year-old children. Figure 2 illustrates the yearly distribution of RSVH in 0–5-year-olds from 2008 to 2020. The average annual incidence of RSVH in 0–1-year-old children was 0.9518% (2009) to 1.7113% (2020); in 1–2-year-old children it was 0.1665% (2009) to 0.7487% (2020); in 2–3-year-old children 0.2018% (2008) to 0.9532% (2020); in 3–4-year-old children 0.0476% (2008) to 0.5924% (2020); and in 4–5-year-old children 0.0197% (2009) to 0.3338% (2020). The RSVH annual incidence was significantly higher in 0–1-year-old children than in 1–5-year-old children.

### 3.2. Monthly Case Number and AAP of RSVH in 2008–2020

**Figure 3:**
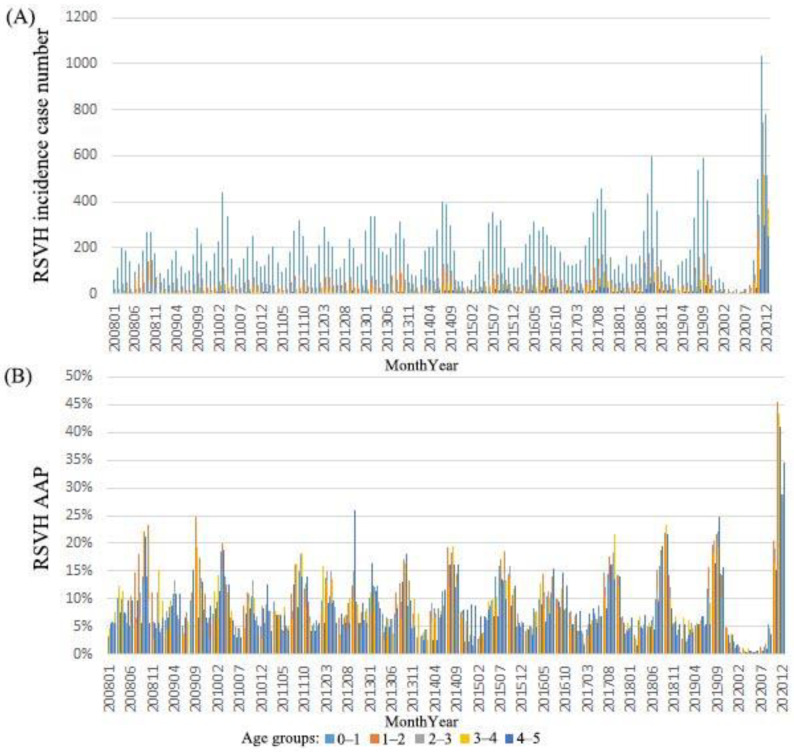
Monthly number (**A**) and AAP (**B**) of RSVHs among 0–5-year-old children. (**A**) Monthly RSVH number included patients classified as having RSVH if they had a hospital admission record in index month/year with one of the following diagnoses at discharge: ICD-9-CM codes: (years 2008–2015) 079.6 (RSV infection), 480.1 (RSV pneumonia), and 466.11 (RSV bronchiolitis); ICD-10-CM codes: (years 2016–2020) B97.4 (RSV as the cause of diseases classified elsewhere), J20.5 (acute bronchitis due to RSV), J21.0 (acute bronchiolitis due to RSV), and J12.1 (RSV pneumonia). (**B**) The RSVH AAP was calculated by monthly RSVH number in index year divided by annual RSVH number in index year. The minimum number of months to accumulate for total AAP to reach 75% was estimated as the RSV epidemic season.

**Figure 4:**
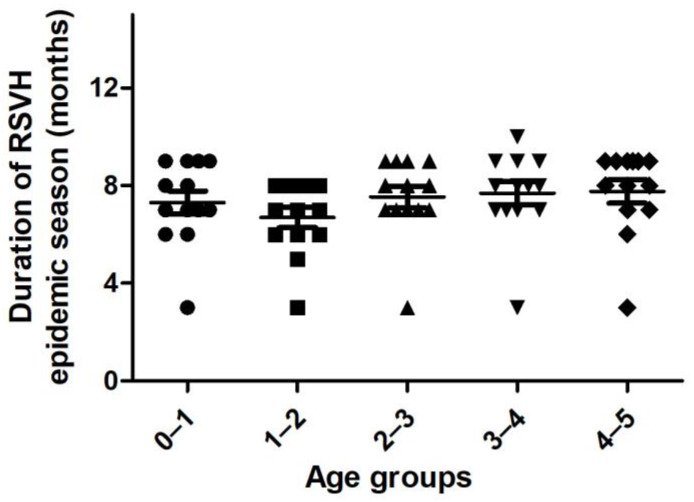
The duration of RSV epidemic season in Taiwan. The RSVH AAP was calculated by monthly RSVH number in index year divided by annual RSVH number in index year. The minimum number of months to accumulate for total AAP to reach 75% was estimated as the RSV epidemic season. The duration of RSV epidemic season in different-aged children was calculated for each year. The symbols indicated that the duration of RSV epidemic season among 0–5-year-old children in 2008-2020.

The monthly RSVH throughout the study period is displayed in Figure 3A (the detailed number is shown in Table S2). RSVH peaked in February–May and July–August from 2008 to 2019. RSVHs were rare until autumn 2020. However, a significant RSVH surge was observed after September 2020 lasting to the end of 2020 in all age groups. In November 2020, there were 1035 cases in age group 0–1 years, 617 cases in age group 1–2 years, 743 cases in age group 2–3 years, 515 cases in age group 3–4 years, and 296 cases in age group 4–5 years. For each year, we utilized AAP 75% to estimate the RSV epidemic season. There was a 13-year follow-up for these 5 groups, with most years having 2 or 3 RSV epidemic seasons each (Figure 3B). Among these 5 groups, only 1 RSV epidemic season was found in 2009/2015 in age group 0–1 years; in 2008/2012/2015 in age group 1–2 years; in 2012 in age group 2–3 years; in 2012/2014/2015 in age group 3–4 years; and in 2014/2018 in age group 4–5 years. October to December 2020 had a high incidence of RSVH, and all age groups had a single RSV epidemic season in the year 2020. The duration of the RSV epidemic season in Taiwan for all age groups was around 7 months (7.31 months (95%CI: 6.28–8.34) in age group 0–1 years, 6.69 months (95%CI: 5.79–7.60) in age group 1–2 years, 7.54 months (95%CI: 6.56–8.51) in age group 2–3 years, 7.69 months (95%CI: 6.66–8.72) in age group 3–4 years, and 7.77 months (95%CI: 6.72–8.82) in age group 4–5 years) (Figure 4). The duration of the RSV epidemic season did not vary significantly across groups.

### 3.3. RSVH Risk among Different Birth Months

**Figure 5:**
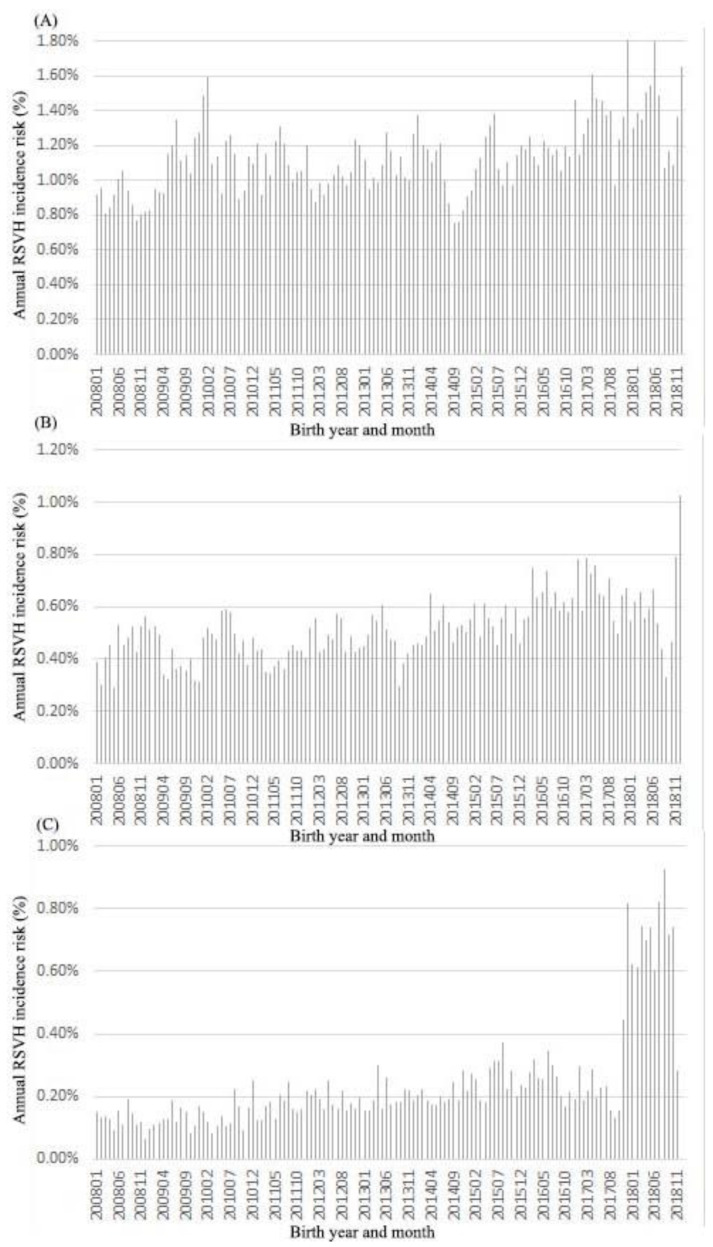
RSVH risk among different birth months for one- (**A**), two- (**B**), and three- (**C**) year-olds. According to the birth month and year, we checked the RSVH rate of children born between January 2008 (200801) and December 2018 (201812) every year until 3 years old.

Figure 5 shows the yearly RSV infection risk for children born between January 2008 (200801) and December 2018 (201812). RSVH incidence rates in the first year varied from 0.77% (October 2008) to 1.80% (June 2018). RSVH incidence rates decreased in the second year, ranging from 0.29% (May 2008) to 1.03% (December 2018). Third-year RSVH incidence rates were lower in children born between January 2008 and October 2017; however, there was a significant rise in children born between November 2017 and December 2018, with a peak of 0.93% in August 2018.

## 4. Discussion

The main finding of this NHIRD-based study was that the risk of RSVH in infants was significantly higher than in young children in real-world practice in Taiwan. The RSVH annual risk also reduced as age increased. In most years, 0–5-year-old children faced 2 or 3 RSV epidemic seasons every year in Taiwan during the 13-year follow-up period. To the best of our knowledge, this is the longest and largest real-world study of RSVH risk in Taiwan in recent years. We also observed an RSVH surge in Taiwan from September to December 2020 among children aged 0–5. 

In temperate-climate countries, the RSV season occurs in late autumn or winter, and babies, particularly those less than 6 months old, have a higher RSVH incidence [25,26]. Unlike countries in temperate zones, we found that the RSV seasons of infants were in Feb–May and Jul–Nov by analyzing the 2007–2009 national claim database [14]. Our study also demonstrated that the RSV peaks during the years 2008–2013 in Taiwan were in the spring and fall. RSV cases peaked in Taiwan in late spring to early autumn from 2014 to 2019 (non-outbreak period). After the World Health Organization’s declaration of COVID-19 as a worldwide pandemic in early 2020, social activities in Taiwan were banned in order to prevent infection via social distancing, mask wearing, and handwashing. Since COVID-19 control has hampered the propagation of RSV, the regulation epidemic peaks were missing in 2020, notably in Australia [23]. Elena et al. also reported that a drastic reduction in RSVH was observed in the Northern and Southern hemispheres [27]. The masks, hand cleaning, and quarantines were also protection tools for other respiratory virus infections, and these regulations have been implemented in Taiwan since early 2020 to prevent COVID-19 spreading. Clinciu et al. found that there were no severe influenza cases in Taiwan, using the Taiwan National Infectious Disease Statistics System [28]. They also found that no cause of severe meningococcal meningitis was reported from February 2020 to March 2021. These results indicate that COVID-19 prevention and control can lower various respiratory virus infections and that the reduction in these infections in the early and middle of 2020 was similar to that in RSV infection. However, at the end of 2020 in Australia, there was a strange rise in the number of RSV cases. In another study in China, RSV positive cases were significantly increasing (*p* < 0.001) from 7.6% (402) to 9.6% (288) and 13.8% (415) in 2019, 2020, and 2021, respectively [29]. Among these RSV-positive patients, most were under two years old and especially under one year old. The rising RSV incidence during winter 2020–21 was probably caused by young children without natural immunity to RSV due to social distancing [30]. In this study, we also found that yearly case numbers in 2020 were 2.25, 1.74, and 1.75 times higher than the seasonal peak in 2017, 2018, and 2019, respectively. Our results were consistent with previous research. 

According to Aihara et al.’s report, RSV-associated hospitalization was highest in young infants (below three months of age) and in those born around the RSV season [31]. In our previous study, we found that the incidence of RSVH started to increase after 3 months and peaked between 7 and 9 months of chronologic age, and the RSV seasons of infants were in February–May and July–November [14]. When combining these two datasets, the high-RSVH-risk birth months were May–July and October–December. In this study, infants born in May to July and October to December had a higher RSVH risk than other birth months (Figure 3). With global data on RSV community mortality, You et al. found that 1 death was attributable to RSV in every 50 deaths in all 0–5-year-old children and in 28 deaths in 1–6-month-old children [32]. In acute lower respiratory infection hospitalization death, there are high associations with RSV etiology. RSVH is not only associated with health risk but also with high medical expenditure. Several studies demonstrated that RSV etiology is associated with higher hospitalization costs due to the longer length of hospitalization and higher ICU usage as compared with other etiologies in children with bronchiolitis [33]. RSV prophylaxis regimen (palivizumab) has been reimbursed in Taiwan for infants of gestational age (GA) ≤ 28 complete weeks or for those born at 29–35 GA with bronchopulmonary dysplasia (BPD) since December 2010. Because the numbers of preterm and severe disease patients are stable and the budget is easy to predict, the National Health Insurance Administration in Taiwan increased indications for RSV prophylaxis regimen to infants of GA ≤ 33 complete weeks or with congenital heart disease. Another single-intramuscular-injection RSV monoclonal antibody (Nirsevimab) used for RSV prophylaxis was reported to significantly lower the incidence of RSV-associated lower respiratory tract infection and RSV-associated hospitalizations in healthy infants [34]. Our results could help to increase focus on both high-risk and normal infant populations, which may improve the healthcare burden and also indicate a direction for authorities to develop future prevention strategies.

Although the analyses of the NHIRD data have provided several benefits, such data still have limitations due to the NHIRD design. First, the goals of NHIRD have led to a lack of information about self-payment for medications, lab test results, and patient information (height, weight, etc.). It is also not possible to measure the severity of the disease. Second, this study design was strictly based on the ICD system, so coding errors, misclassifications, or differences between hospitals and physicians could have affected these results, which could make the proportions of our subjects incorrect. Because the National Health Insurance Administration takes time to update their claims database, the one- to one-and-a-half-year time lag to obtain the NHIRD is another study limitation. At the time we applied for the NHIRD, only data for 1996–2020 was available. This is the reason we used the year 2020 as the latest year to analyze the COVID-19 pandemic, and we were unable to observe the impact of the COVID-19 pandemic in 2021–2022.

## 5. Conclusions

In this study, we found RSVH-consistent peaks in February–May and July–August. The 2020 RSV outbreak was discovered at the end of 2020. Infants born in May–July and October–December showed a higher RSVH risk than in other birth months.

## Data Availability

The data underlying this study belong to the National Health Insurance Research Database (NHIRD) of Taiwan and cannot be made publicly available due to legal restrictions. However, the data are available through formal application to the Health and Welfare Data Science Centre at the Ministry of Health and Welfare, Taiwan (https://dep.mohw.gov.tw/DOS/np-2500-113.html)(accessed on 19 January 2020) and require a signed affirmation regarding data confidentiality. The authors have no special privilege of access to the database.

## Supplementary Materials

The following supporting information can be downloaded at: https://www.mdpi.com/article/10.3390/children10040629/s1, Table S1: Annual incidence of RSVH among 0–5-year-old children; Table S2: Monthly AAP of RSVHs among 0–5-year-old children; Table S3: RSVH risk among different birth months for one- (A), two- (B), and three- (C) year-olds.
