# Supplementary material for: Respiratory Syncytial Virus Outbreak in Infants and Young Children during COVID-19 Pandemic in Taiwan
Source: Children (Basel). 2023 Mar 28;10(4):629. doi: 10.3390/children10040629 (PMC10137034; doi:10.3390/children10040629)
Supplement: Supplementary file 1 [file children-10-00629-s001.zip › children-2202763-supplementary.pdf]

Table S1: Annual incidence of RSVH among 0–5-year-old children

	0–1	1–2	2–3	3–4	4-5
2008	1.0300%	0.3066%	0.2018%	0.0476%	0.0206%
2009	0.9518%	0.1665%	0.2322%	0.0644%	0.0197%
2010	1.5297%	0.2863%	0.2701%	0.1205%	0.0454%
2011	1.1433%	0.2782%	0.2304%	0.0688%	0.0311%
2012	0.9774%	0.2630%	0.2604%	0.0793%	0.0246%
2013	1.4852%	0.2958%	0.3192%	0.1081%	0.0297%
2014	1.2300%	0.2849%	0.3339%	0.1298%	0.0614%
2015	1.0986%	0.2558%	0.2859%	0.1246%	0.0726%
2016	1.3196%	0.3625%	0.3542%	0.1490%	0.0783%
2017	1.5509%	0.3949%	0.4092%	0.1944%	0.0904%
2018	1.5952%	0.4370%	0.4589%	0.1808%	0.1082%
2019	1.6729%	0.3924%	0.3906%	0.1553%	0.0661%
2020	1.7113%	0.7487%	0.9532%	0.5924%	0.3338%

Table S2: Monthly AAP of RSVHs among 0–5-year-old children

AAP					
Birth months/Age	0–1	1–2	2–3	3–4	4-5
200801	3.31%	0.00%	4.64%	4.76%	5.77%
200802	5.90%	0.00%	5.57%	7.62%	5.77%
200803	10.25%	0.00%	10.44%	12.38%	7.69%
200804	9.89%	0.00%	11.14%	11.43%	7.69%
200805	7.40%	0.00%	5.57%	9.52%	9.62%
200806	5.12%	10.49%	5.34%	7.62%	9.62%
200807	6.63%	14.71%	6.73%	5.71%	9.62%
200808	9.63%	18.15%	11.14%	6.67%	5.77%
200809	13.92%	22.07%	12.76%	10.48%	21.15%
200810	14.03%	23.32%	9.74%	5.71%	5.77%
200811	9.11%	11.27%	5.80%	2.86%	5.77%
200812	4.81%	0.00%	11.14%	15.24%	5.77%
200901	3.91%	0.89%	4.67%	9.63%	6.52%
200902	6.10%	3.85%	7.71%	4.44%	6.52%
200903	8.63%	4.44%	9.74%	9.63%	8.70%
200904	10.82%	5.62%	13.39%	10.37%	10.87%
200905	7.02%	2.66%	6.49%	6.67%	10.87%
200906	5.29%	3.85%	3.65%	3.70%	6.52%
200907	5.81%	7.69%	3.45%	5.93%	0.00%
200908	9.72%	11.24%	9.53%	5.93%	15.22%
200909	16.51%	24.85%	18.66%	19.26%	6.52%
200910	12.37%	17.46%	13.79%	11.85%	13.04%
200911	8.06%	10.95%	6.09%	4.44%	6.52%
200912	5.75%	6.51%	2.84%	8.15%	8.70%
201001	7.23%	5.59%	4.59%	11.46%	8.33%
201002	9.39%	6.82%	10.28%	14.23%	11.46%
201003	18.45%	19.93%	15.05%	18.58%	18.75%
201004	13.92%	12.59%	10.64%	11.07%	12.50%
201005	6.32%	6.64%	5.87%	7.91%	6.25%
201006	3.41%	5.07%	4.22%	2.77%	3.13%
201007	4.78%	4.72%	4.77%	2.77%	3.13%
201008	6.36%	8.74%	4.59%	4.74%	7.29%
201009	8.65%	11.19%	10.83%	9.49%	8.33%

201010	10.47%	8.74%	13.39%	10.28%	7.29%
201011	6.03%	5.94%	6.97%	3.95%	5.21%
201012	4.99%	4.02%	8.81%	2.77%	8.33%
201101	5.74%	3.97%	9.07%	7.80%	12.68%
201102	7.93%	4.38%	7.78%	4.26%	4.23%
201103	9.47%	7.72%	6.91%	7.80%	7.04%
201104	6.35%	7.10%	4.32%	7.09%	7.04%
201105	4.53%	4.18%	3.67%	8.51%	7.04%
201106	5.27%	4.80%	4.10%	4.96%	4.23%
201107	8.59%	10.86%	7.78%	6.38%	12.68%
201108	12.74%	16.28%	12.96%	16.31%	8.45%
201109	14.93%	15.45%	18.14%	18.44%	14.08%
201110	11.62%	11.90%	12.74%	8.51%	14.08%
201111	7.65%	9.39%	6.91%	5.67%	4.23%
201112	5.18%	3.97%	5.62%	4.26%	4.23%
201201	6.21%	3.35%	5.17%	2.56%	5.56%
201202	9.81%	7.92%	10.12%	16.03%	5.56%
201203	13.69%	12.50%	15.08%	13.46%	9.26%
201204	10.56%	12.50%	15.08%	13.46%	9.26%
201205	9.67%	7.04%	8.47%	3.21%	5.56%
201206	5.09%	6.51%	3.51%	3.21%	7.41%
201207	5.42%	6.69%	4.55%	7.05%	5.56%
201208	7.06%	9.33%	4.34%	10.26%	5.56%
201209	11.31%	12.32%	14.88%	9.62%	25.93%
201210	9.39%	9.15%	6.20%	8.97%	5.56%
201211	5.65%	7.57%	4.75%	3.85%	9.26%
201212	6.12%	5.11%	7.85%	8.33%	5.56%
201301	10.15%	7.14%	9.23%	11.11%	16.39%
201302	12.31%	7.70%	12.10%	10.58%	11.48%
201303	12.35%	6.44%	9.53%	7.41%	8.20%
201304	7.29%	4.34%	3.93%	2.65%	4.92%
201305	6.63%	3.50%	6.35%	6.35%	4.92%
201306	6.34%	6.30%	6.35%	3.70%	0.00%
201307	7.29%	11.06%	6.81%	8.99%	8.20%
201308	9.56%	12.75%	9.38%	6.35%	13.11%
201309	11.43%	17.09%	14.07%	16.40%	18.03%
201310	8.79%	13.31%	9.68%	8.99%	9.84%

201311	4.80%	7.42%	6.20%	10.05%	4.92%
201312	3.04%	2.94%	6.35%	7.41%	0.00%
201401	3.26%	2.78%	3.45%	4.07%	2.68%
201402	4.49%	2.96%	4.43%	2.59%	0.00%
201403	7.63%	7.83%	9.24%	5.93%	2.68%
201404	8.32%	7.30%	7.39%	6.67%	2.68%
201405	8.36%	6.26%	6.65%	7.78%	7.14%
201406	11.34%	8.87%	8.25%	10.00%	11.61%
201407	16.40%	19.30%	16.26%	12.22%	16.07%
201408	15.83%	18.26%	16.38%	19.63%	16.07%
201409	12.20%	14.43%	12.81%	11.48%	16.07%
201410	7.59%	7.13%	7.76%	7.41%	8.04%
201411	2.33%	2.26%	3.82%	5.93%	8.04%
201412	2.24%	2.61%	3.57%	6.30%	8.93%
201501	1.58%	2.50%	3.27%	6.15%	8.70%
201502	2.71%	1.78%	1.72%	3.24%	6.83%
201503	3.79%	3.21%	3.96%	3.88%	6.83%
201504	6.50%	6.06%	5.85%	9.39%	8.07%
201505	8.72%	8.56%	9.64%	10.03%	6.83%
201506	13.91%	8.38%	10.15%	10.36%	6.83%
201507	16.03%	17.11%	12.05%	9.39%	13.66%
201508	13.37%	18.54%	14.46%	13.27%	9.94%
201509	14.41%	14.26%	15.83%	13.59%	8.70%
201510	8.94%	10.70%	9.64%	11.97%	12.42%
201511	5.01%	6.06%	7.40%	4.85%	5.59%
201512	5.01%	2.85%	6.02%	3.88%	5.59%
201601	4.27%	4.20%	4.29%	4.82%	4.46%
201602	5.20%	3.95%	5.05%	4.82%	3.47%
201603	8.28%	7.41%	7.58%	7.72%	4.95%
201604	9.85%	8.02%	10.35%	12.86%	8.91%
201605	12.20%	14.57%	11.24%	8.36%	5.94%
201606	10.47%	9.63%	11.36%	10.29%	7.43%
201607	11.28%	13.95%	9.85%	8.04%	15.35%
201608	9.97%	10.00%	9.85%	8.36%	9.41%
201609	8.08%	8.64%	8.59%	11.90%	14.85%
201610	7.97%	8.02%	8.33%	10.61%	12.38%
201611	7.01%	7.53%	7.83%	5.47%	5.45%

201612	5.43%	4.07%	5.68%	6.75%	7.43%
201701	4.32%	4.30%	3.55%	5.28%	7.85%
201702	4.32%	2.33%	3.66%	2.06%	1.57%
201703	4.57%	3.60%	5.48%	3.21%	7.33%
201704	5.27%	5.35%	4.84%	6.19%	8.38%
201705	7.42%	5.81%	6.34%	6.42%	5.76%
201706	8.68%	5.58%	6.88%	5.96%	6.81%
201707	12.51%	14.65%	12.04%	10.55%	8.38%
201708	14.41%	17.56%	13.33%	14.91%	16.23%
201709	16.17%	17.67%	18.28%	21.56%	13.61%
201710	12.83%	14.19%	14.30%	12.39%	14.14%
201711	5.69%	6.51%	6.77%	5.73%	5.76%
201712	3.80%	2.44%	4.52%	5.73%	4.19%
201801	4.63%	3.73%	5.00%	3.69%	6.64%
201802	3.42%	3.16%	2.20%	2.70%	1.66%
201803	6.06%	3.62%	5.09%	6.88%	4.98%
201804	4.78%	3.95%	5.49%	3.44%	7.05%
201805	4.85%	4.75%	4.60%	5.65%	4.98%
201806	6.10%	4.07%	6.79%	5.16%	4.56%
201807	10.00%	15.14%	11.19%	10.07%	9.54%
201808	15.91%	18.87%	17.58%	18.67%	19.50%
201809	21.90%	20.68%	19.98%	23.34%	21.58%
201810	13.30%	14.35%	12.19%	10.07%	8.30%
201811	5.51%	5.76%	5.39%	6.88%	5.81%
201812	3.53%	1.92%	4.50%	3.44%	5.39%
201901	2.84%	2.46%	4.81%	6.51%	5.48%
201902	2.35%	1.77%	2.78%	6.21%	3.42%
201903	4.37%	3.27%	5.57%	4.73%	4.11%
201904	5.12%	4.64%	5.19%	5.62%	5.48%
201905	5.47%	4.91%	6.08%	5.92%	6.85%
201906	6.97%	5.05%	5.19%	4.14%	5.48%
201907	11.76%	15.69%	10.38%	9.17%	5.48%
201908	19.08%	19.65%	20.51%	18.34%	16.44%
201909	21.04%	21.56%	22.03%	19.53%	24.66%
201910	14.50%	14.19%	10.38%	11.54%	15.75%
201911	4.30%	4.91%	4.81%	4.44%	3.42%
201912	2.20%	1.91%	2.28%	3.85%	3.42%

202001	2.41%	1.03%	1.23%	0.59%	1.67%
202002	1.78%	1.40%	0.67%	0.25%	0.42%
202003	0.82%	1.18%	0.56%	0.59%	0.42%
202004	0.33%	0.44%	0.50%	1.09%	0.69%
202005	0.74%	0.44%	0.39%	0.25%	0.42%
202006	0.41%	0.59%	0.45%	0.67%	0.56%
202007	0.78%	1.40%	0.50%	0.59%	0.56%
202008	1.34%	1.91%	1.51%	1.18%	0.83%
202009	5.42%	4.42%	4.60%	3.87%	3.61%
202010	18.61%	20.47%	19.11%	16.47%	15.14%
202011	38.45%	45.43%	41.65%	43.28%	41.11%
202012	28.90%	21.28%	28.81%	31.18%	34.58%

Table S3: RSVH risk among different birth months for one- (A), two- (B), and three- (C) year-olds.

Birth months	First year	Second year	Third year
200801	0.92%	0.39%	0.15%
200802	0.96%	0.30%	0.13%
200803	0.81%	0.41%	0.14%
200804	0.85%	0.45%	0.13%
200805	0.92%	0.29%	0.09%
200806	1.01%	0.53%	0.16%
200807	1.05%	0.45%	0.11%
200808	0.95%	0.48%	0.19%
200809	0.86%	0.53%	0.15%
200810	0.77%	0.43%	0.11%
200811	0.81%	0.53%	0.12%
200812	0.82%	0.56%	0.06%
200901	0.83%	0.52%	0.10%
200902	0.95%	0.53%	0.11%
200903	0.93%	0.49%	0.11%
200904	0.93%	0.34%	0.13%
200905	1.16%	0.32%	0.13%
200906	1.21%	0.44%	0.19%
200907	1.35%	0.36%	0.12%
200908	1.12%	0.37%	0.16%
200909	1.15%	0.36%	0.15%
200910	1.04%	0.40%	0.08%
200911	1.25%	0.32%	0.11%
200912	1.28%	0.31%	0.17%
201001	1.49%	0.48%	0.15%
201002	1.59%	0.52%	0.12%
201003	1.10%	0.50%	0.08%
201004	1.14%	0.48%	0.11%
201005	0.92%	0.59%	0.14%
201006	1.23%	0.59%	0.10%
201007	1.26%	0.58%	0.11%
201008	1.15%	0.50%	0.22%
201009	0.90%	0.42%	0.17%
201010	0.95%	0.47%	0.09%
201011	1.14%	0.38%	0.17%

201012	1.10%	0.48%	0.25%
201101	1.21%	0.43%	0.12%
201102	0.92%	0.44%	0.12%
201103	1.15%	0.35%	0.17%
201104	1.03%	0.34%	0.18%
201105	1.23%	0.37%	0.13%
201106	1.31%	0.39%	0.20%
201107	1.21%	0.36%	0.19%
201108	1.09%	0.43%	0.24%
201109	1.00%	0.46%	0.16%
201110	1.04%	0.43%	0.15%
201111	1.05%	0.43%	0.16%
201112	1.21%	0.41%	0.22%
201201	0.95%	0.52%	0.20%
201202	0.88%	0.56%	0.22%
201203	0.98%	0.43%	0.19%
201204	0.92%	0.44%	0.16%
201205	0.98%	0.49%	0.25%
201206	1.03%	0.47%	0.17%
201207	1.09%	0.58%	0.16%
201208	1.02%	0.56%	0.22%
201209	0.98%	0.43%	0.15%
201210	1.05%	0.49%	0.18%
201211	1.23%	0.43%	0.16%
201212	1.20%	0.44%	0.20%
201301	1.12%	0.45%	0.15%
201302	0.95%	0.49%	0.16%
201303	1.02%	0.57%	0.19%
201304	1.00%	0.55%	0.30%
201305	1.09%	0.60%	0.16%
201306	1.28%	0.52%	0.26%
201307	1.17%	0.47%	0.18%
201308	1.03%	0.47%	0.18%
201309	1.14%	0.30%	0.18%
201310	1.02%	0.39%	0.22%
201311	1.00%	0.42%	0.22%
201312	1.27%	0.45%	0.19%

201401	1.37%	0.46%	0.20%
201402	1.20%	0.45%	0.22%
201403	1.18%	0.48%	0.19%
201404	1.11%	0.65%	0.18%
201405	1.17%	0.51%	0.17%
201406	1.21%	0.55%	0.20%
201407	1.00%	0.60%	0.18%
201408	0.87%	0.54%	0.19%
201409	0.76%	0.46%	0.24%
201410	0.77%	0.52%	0.19%
201411	0.83%	0.53%	0.28%
201412	0.91%	0.50%	0.22%
201501	0.95%	0.55%	0.27%
201502	1.07%	0.61%	0.26%
201503	1.13%	0.48%	0.19%
201504	1.25%	0.61%	0.18%
201505	1.32%	0.56%	0.29%
201506	1.39%	0.52%	0.31%
201507	1.07%	0.45%	0.31%
201508	0.98%	0.56%	0.37%
201509	1.10%	0.61%	0.22%
201510	0.98%	0.50%	0.28%
201511	1.14%	0.60%	0.20%
201512	1.20%	0.46%	0.24%
201601	1.18%	0.55%	0.23%
201602	1.25%	0.56%	0.28%
201603	1.14%	0.75%	0.32%
201604	1.09%	0.64%	0.26%
201605	1.23%	0.65%	0.25%
201606	1.19%	0.74%	0.34%
201607	1.15%	0.59%	0.30%
201608	1.18%	0.65%	0.27%
201609	1.06%	0.58%	0.20%
201610	1.19%	0.62%	0.17%
201611	1.14%	0.58%	0.22%
201612	1.47%	0.63%	0.19%
201701	1.15%	0.78%	0.29%

201702	1.27%	0.59%	0.19%
201703	1.36%	0.79%	0.22%
201704	1.61%	0.73%	0.29%
201705	1.47%	0.76%	0.20%
201706	1.46%	0.65%	0.23%
201707	1.37%	0.64%	0.23%
201708	1.40%	0.71%	0.16%
201709	0.97%	0.55%	0.13%
201710	1.24%	0.50%	0.15%
201711	1.36%	0.64%	0.45%
201712	1.81%	0.67%	0.82%
201801	1.30%	0.55%	0.62%
201802	1.39%	0.62%	0.61%
201803	1.35%	0.65%	0.74%
201804	1.51%	0.56%	0.70%
201805	1.55%	0.59%	0.74%
201806	1.80%	0.66%	0.61%
201807	1.49%	0.54%	0.82%
201808	1.07%	0.44%	0.93%
201809	1.17%	0.33%	0.72%
201810	1.09%	0.47%	0.74%
201811	1.37%	0.79%	0.28%
201812	1.65%	1.03%	0.00%
